# Pollution of Streams

**Published:** 1917-04

**Authors:** 


**Pollution of Streams.** The N. Y. Supreme Court has upheld damages awarded by a lower court against the city of Batavia for pollution of Tonawanda Creek. (Pub. Health Reports). This is a radical decision when we consider the geography. Tonawanda Creek is a winding, rather sluggish stream, scarcely available or needed to supply potable water to any town. With the exception of Batavia, it passes no city till it reaches the Niagara River between the Tonawandas. Logically interpreted, this decision means that there shall be no rural pollution of any stream.

